# A South African national database in cardiothoracic surgery

**Published:** 2010-06

**Authors:** Anthony Linegar, Francis Smit, Andrie Stroebel, Elizabeth Schaafsma

**Affiliations:** Department Cardiothoracic Surgery, University of the Free State, Bloemfontein, South Africa; Department Cardiothoracic Surgery, University of the Free State, Bloemfontein, South Africa; Department Cardiothoracic Surgery, University of the Free State, Bloemfontein, South Africa; South African Heart Association, Department of Cardiology, Tygerberg Hospital, Cape Town, South Africa

**Keywords:** national database, cardiothoracic surgery, South Africa

## Abstract

**Summary:**

This article aims to update South African cardiothoracic surgeons on the developmental progress of the national database in cardiac and thoracic surgery and to encourage participation in this most important endeavour.

## The role of databases in evidence-based cardiothoracic surgery

Cardiothoracic surgery in the academic units of South Africa faces challenges on two fronts; to keep pace with international developments in cardiothoracic surgery and simultaneously to survive the trials of a healthcare service in crisis.^1^ Both involve the need to meet the growing demand for service from the profession and the public, and to increase the evidence basis in clinical decision making.^2^

Evidence-based medicine (EBM) has been described as the judicious combination of best external evidence, integrated with the clinician’s clinical experience and insight.^3^ Its philosophy is to minimise intuitive decision making and to replace anecdotal dogma with a clear treatment plan individualised to the patient’s circumstances, without substituting clinical insight for inflexibility.

Databases perform two broad functions; one is specifically designed to collect data in answer to a specific research question involving a series of patients, and the other is a prospective continual recording of a wide range of data on all patients treated by a speciality, for later use in clinical and healthcare systems research. Databases therefore provide the basis on which to convert information into knowledge that guides clinical practice.^4^

Analysis of accurately collected data allows the interpretation of outcomes of treatment, provides information for prognostication and identifies factors that influence outcome in subsets of patients or operations. Trends, strengths and weaknesses that emerge from this process will facilitate strategic planning and advocacy with health policy makers in resource allocation, training requirements and speciality development.^5^,^6^

A national database is therefore an important asset to cardiothoracic surgery in South Africa.

## Design features of the database

The South African National Cardiothoracic Surgery database was written by eMD® on an IBM Lotus platform and uses XML and Java Scripting. eMD® will continue to provide programming assistance and maintenance on code-related issues.

Data entry fields are based on the Society for Thoracic Surgeons (STS) database (SND) with necessary adaptations for local relevance.^7^ It is designed on the principle of minimal free-hand data entry, using pull-down windows to select predetermined options, but where appropriate, free-text data entry fields, e.g. for operation notes, are included. It has the ability to export information on selected subsets of patients for deeper statistical analysis.

## Field description

Data entry begins with patient demographics. The patient’s national identity (ID) number prevents duplication of patient files. In the case of a paediatric patient without a national ID number, the date of birth serves this function. A unique, chronological identification number is automatically allocated to each patient for the filing of hard-copy patient folders.

Sections on history and clinical examination follow this, and include risk factors in cardiac and thoracic surgery. Thereafter come sections on special examinations such as electrocardiogram, lung functions, echocardiography and radiological investigations. The intention is to add digital radiological images in the future. Finally, it records outcome data, complications, mortality and discharge data including medications and follow-up plans.

The program also contains a complete practice management system with tariff and ICD 10 codes and software to interface with the medical aid industry. It provides accounting details for tracking debtors, performing audit trails and monthly aged analysis.

## Report generation

Generation of clinical reports is individualised to specific user requirements using any combination of field variables. It can, for example be structured to print a patient summary for report back to a referring colleague, or alternatively create print outs for a monthly or annual clinical audit.

## Security and anonymity

Registered users gain access to their own data by password. Data can either be stored on their PC, or a server can be accessed via an internet connection. Data are stored in an encrypted form on a central server with industry standard backup and firewall protection. Data protection is an ethical priority in database management and a university ethics board (UFS Ethics Committee ETOV S46/08) has approved the protocols used. Access to any data other than the users’ ‘own’ for research purposes is subject to authorisation on application to the working group committee of the South African Heart Association. Anonymity of doctors and patients will be protected in any extract of data.

## Conclusion

This is a powerful tool for clinical development and advocacy that has the potential to serve as a repository of a wide range of patient data, which can be selectively exported for investigation of specific clinical research problems.

The SND was established in 1989 and has since become the gold standard of national databases. Participation in the USA had grown to over 1 500 surgeons in 706 hospitals within five years of its inception.^6^,^8^ Since then it has undergone many improvements and now includes general thoracic surgery. The South African National Cardiothoracic Surgery database is based on the SND because of a need for an already proven database that could be easily adapted to South African requirements and that would facilitate a common ground for international communication regarding the data collected and the definitions applied to it.

It is hoped that the cardiothoracic surgery community in South Africa will participate enthusiastically, as the long-term benefits to South African cardiothoracic surgery and the public are significant. Surgeons wishing to obtain access to the database can contact Mrs Elizabeth Schaafsma on e-mail elizabeth@vodamail.co.za.

## References

[1] Gevers W (2009). Clinical Research in South Africa: a core asset under pressure.. Lancet.

[2] Lee JS, Urschel DM, Urschel JD (2000). Is general thoracic surgery practice evidence based?. Ann Thorac Surg.

[3] Sackett DL, Rosenberg WMC, Gray JAM, Haynes RB, Richardson WS (1996). Evidence based medicine: what it is and what it isn’t.. Br Med J.

[4] Ferguson M, Ferguson M (2007). Introduction. Difficult Decisions in Thoracic Surgery: An Evidence-Based Approach..

[5] Anderson RP (1994). First publications from the Society of Thoracic Surgeons national database.. Ann Thorac Surg.

[6] Clark RE (1995). The STS cardiac surgery national database: an update.. Ann Thorac Surg.

[7] The Society of Thoracic Surgeons General Thoracic Surgery Database Data Collection Form Version 2.06.2004.. http://www.ctsnet.org/file/ThoracicDCFV2_06_Nonannotated.pdf..

[8] Ferguson TB, Dziuban SW, Edwards FH, Eiken MC, Shroyer AL, Pairolero PC (2000). The STS national database: current changes and challenges for the new millennium. Committee to establish a national database in cardiothoracic surgery, the Society of Thoracic Surgeons.. Ann Thorac Surg.

